# The role of WNT/β-catenin signaling pathway in melanoma epithelial-to-mesenchymal-like switching: evidences from patients-derived cell lines

**DOI:** 10.18632/oncotarget.9232

**Published:** 2016-05-09

**Authors:** Daniela Kovacs, Emilia Migliano, Luca Muscardin, Vitaliano Silipo, Caterina Catricalà, Mauro Picardo, Barbara Bellei

**Affiliations:** ^1^ Laboratory of Cutaneous Physiopathology and Integrated Center of Metabolomics Research, San Gallicano Dermatologic Institute, IRCCS, Rome, Italy; ^2^ Department of Plastic and Reconstructive Surgery, San Gallicano Dermatologic Institute, IRCCS, Rome, Italy; ^3^ Dermatopathological Laboratory, San Gallicano Dermatologic Institute, IRCCS, Rome, Italy; ^4^ Department of Oncologic Dermatology, San Gallicano Dermatologic Institute, IRCCS, Rome, Italy

**Keywords:** WNT/β-catenin, melanoma heterogeneity, proliferation, metastases

## Abstract

Deregulations or mutations of WNT/β-catenin signaling have been associated to both tumour formation and progression. However, contradictory results concerning the role of β-catenin in human melanoma address an open question on its oncogenic nature and prognostic value in this tumour. Changes in WNT signaling pathways have been linked to phenotype switching of melanoma cells between a highly proliferative/non-invasive and a slow proliferative/metastatic condition. We used a novel panel of cell lines isolated from melanoma specimens, at initial passages, to investigate phenotype differences related to the levels and activity of WNT/β-catenin signaling pathway. This *in vitro* cell system revealed a marked heterogeneity that comprises, in some cases, two distinct tumour-derived subpopulations of cells presenting a different activation level and cellular distribution of β-catenin. In cells derived from the same tumor, we demonstrated that the prevalence of LEF1 (high β-catenin expressing cells) or TCF4 (low β-catenin expressing cells) as β-catenin partner for DNA binding, is associated to the expression of two distinct profiles of WNT-responsive genes. Interestingly, melanoma cells expressing relative low level of β-catenin and an invasive markers signature were associated to the TNF-α-induced pro-inflammatory pathway and to the chemotherapy resistance, suggesting that the co-existence of melanoma subpopulations with distinct biological properties could influence the impact of chemo- and immunotherapy.

## INTRODUCTION

The WNT/β-catenin pathway is crucial in embryonic development and adult tissue homeostasis, including cell migration, hematopoiesis and wound repair [1]. A large number of studies have linked WNT signaling to almost every major disease, reflecting the importance of developmental pathways in the pathogenesis of adult disease processes [2–3]. The increased β-catenin expression reported in several types of cancers, compared to normal tissue, has implicated this signaling pathway in oncogenesis. Hyperactivation of WNT signaling promotes migration, invasion and proliferation, linking WNT signaling to more aggressive behavior, and worse prognosis [4–7]. Moreover, during the last few decades the involvement of WNT/β-catenin signaling in the formation and maintenance of cancer stem cells has been suggested [8–9]. β-catenin is a multifunctional protein that plays an important role in the cadherin/catenin complex dynamics involved in cell-cell adhesion, the loss of which may lead to tumor invasion and metastasis [10]. On the other hand, β-catenin serves as an obligate transcriptional co-activator through its ability to interact with the T-cell transcription factor (TCF)/lymphoid enhancer binding factor (LEF) family of DNA binding proteins in the nucleus and to promote chromatin remodeling and transcriptional initiation/elongation [11]. Given these critical but distinct functions, it is not surprising that the level of β-catenin is subjected to tight regulation, particularly through GSK3β-dependent phosphorylation of exon3, which plays a key role in controlling proteasomal degradation [8]. Even if the regulation of β-catenin activity occurs mainly at the level of protein degradation and subcellular localization, there is considerable evidence that its activity depends also on the interaction with distinct partners [12] and on the phosphorylation at several different amino acid residues [13–15]. β-catenin is a co-activator for the expression of ubiquitous genes such as c-MYC *(MYC)* [16], Cyclin D1 (*CCND1*) [17], and AXIN2 (*AXIN2*) [18] as well as cell-lineage restricted genes including Microphthalmia-associated Transcription Factor-M (*MITF-M*) [19–20], Dopachrome Tautomerase (*DCT*) [21], POU class homeoboxes and pseudogenes (*POU3F2,* BRN2, *N-OCT-3*) [22] and many others (http://web.stanford.edu/group/nusselab/cgi-bin/wnt/target_genes).

Evidences from melanocyte development, genetic mouse models, and human patients implicate WNT/β-catenin signaling in melanoma pathogenesis [23–28]. Unlike most cancers, where WNT signaling is considered to be a driver of both tumour formation and progression, in human melanoma there are contradictory results and β-catenin does not satisfactorily correspond to the definition of oncogene [29]. In fact, whereas several studies propose that an increased nuclear translocation and activity of β-catenin promote melanoma proliferation [23], others found that elevated levels of nuclear β-catenin correlate with a more favorable prognosis and a less aggressive disease [30]. Furthermore, almost all benign nevi are positive for nuclear β-catenin, and loss of nuclear β-catenin with melanoma progression to metastases has been reported [24]. These observations from patients collectively indicate the possibility that WNT/β-catenin signaling may not be oncogenic in any sense, but rather is required to maintain a homeostatic balance that, when disrupted or lost, can lead to melanoma transformation and progression. Adding complexity to this topic both the pharmacological activation [31–32] and the inhibition [33–34] of WNT/β-catenin pathway have been proposed for melanoma therapy.

It has been suggested that the tumorigenic susceptibility to an ubiquitously expressed gene such as β-catenin could be intimately related to its function in the development of each tissue [35]. In this prospective the peculiarity of β-catenin in the context of melanocyte lineage is due to the fact that β-catenin is involved not only in the control of the transcription of the melanocyte differentiation gene *MITF*, but also in the modulation of its function through direct protein-protein interaction [20].

MITF appears to be an important regulator of melanoma progression since the knock-down of its gene reduces proliferation and increases invasiveness [36–37]. Moreover, the reduction of MITF expression has been associated to cytoskeletal reorganization and to the phenotype switching, a process similar to epithelial-mesenchymal transition (EMT)-like phenotype (because of their neuroectodermal origin, melanoma cells may not undergo classic EMT) [38–39]. The critical role of β-catenin in phenotypic transition is additionally supported by the anti-correlated expression of MITF and WNT5a, an autocrine/paracrine factor known to contribute to β-catenin regulation [39]. Most of the data supporting this biphasic model results from cell lines isolated from patients with different tumor subtype, disease stage or metastatic location. Moreover, the majority of melanoma cell lines usually employed have been subject to prolonged culture passages, leaving them vulnerable to accumulate genetic abnormalities and loose the genetic profile, the pigmentation characteristics and the metastatic capacity of the original tumor [40–41]. In this study, we used melanoma specimens from patients well characterized for disease stage and follow up to isolate low passage melanoma cell lines and we use this tool to investigate the inter- and intra-tumour heterogenity of WNT signaling regulation in melanoma cells.

## RESULTS

### Melanoma cells isolation and characterization

**Figure 1:**
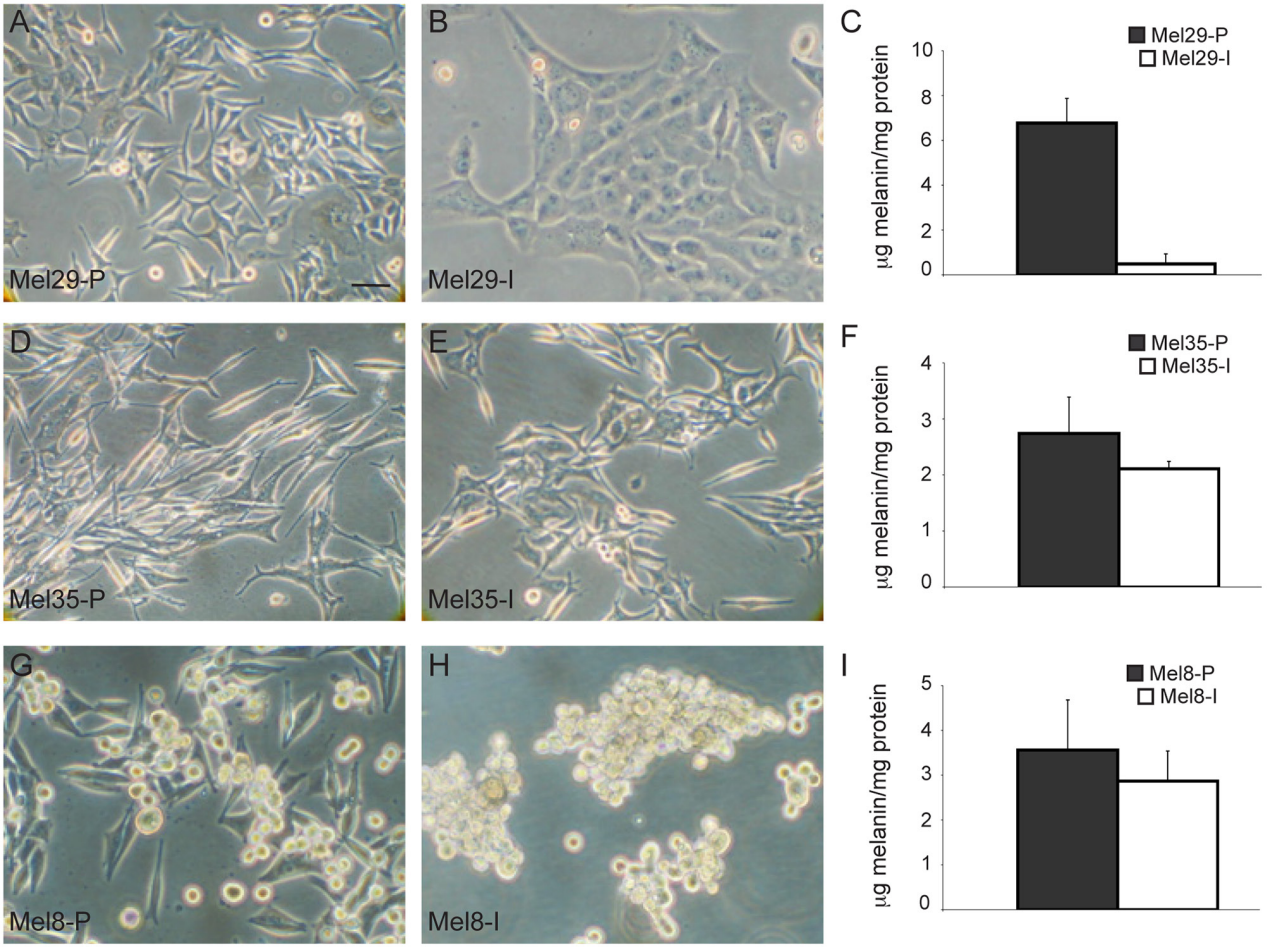
*In vitro* morphological differences of melanoma subpopulations isolated from the same patient Phase contrast microscopic analysis of melanoma subpopulations isolated from the same biopsy evidenced marked morphological differences. In each case, one cell line showed a phenotype similar to that of normal melanocytes with elongated dendritic shape **A, D, G.** and abundant melanin **C, F, I.** and another one displayed a de-differentiated phenotype with low melanin (**C, F, I**) associated to an enlarged morphology similar to epithelial cells **B-E.** or to a rounded shape in the case of Mel8 floating cells **H.** Scale bar: 20 μm.

To advance the knowledge on WNT/β-catenin pathway in melanoma research we collected a panel of new cell lines from fresh primary and metastatic melanoma specimens removed from the skin. Melanoma cells were isolated immediately after the surgical excision and the macroscopical examination of the biopsies as described in material and methods section. Purity of cell cultures was routinely confirmed by immunocytochemical analysis using HMB45, Melan-A and tyrosinase melanocytic differentiation markers (data not shown). To characterize cell lines reflective of the tumor of origin we restricted data collection to low passage *in vitro* cultures (up to passage 12). Freshly isolated melanoma cells were firstly evaluated for their morphology. Phase contrast microscopic analysis showed broad morphological differences highlighting inter-sample heterogeneity (Supplementary Figure 1). Intra-sample heterogeneity was also observed in 3 of the 13 melanoma cell lines successfully stabilized and distinct cell populations obtained from the same tumor lesion were grown independently for comparative studies. In the case of melanoma 29, a primary melanocytic lesion removed from the back of the neck of a 38 year-old male, two cell types were identified based on the different morphology and pigmentation observed after few days of *in vitro* culture: i) one population appeared similar to normal human melanocytes, being mostly dendritic, bipolar or pluripolar, small in shape and highly pigmented, ii) a second population was enlarged, polygonal and epithelioid-like in shape with no evident pigmentation, resembling a de-differentiated morphology (Figure 1A-1C). The two cell types, designed as Mel29-P (proliferative) and Mel29-I (invasive) based on the phenotypical features highlighted in the course of their characterization, were divided using different incubation time with trypsin/EDTA, having the first population the property to be detached faster than the second one. These two melanoma cell types, both carrying the point mutation of V600 (exon 15) in BRAF gene and wild type sequence of hot-spot regions exon 1 and 2 in NRAS gene, were then seeded and cultivated separately. The excised lesion was diagnosed as an ulcerated nodular melanoma with Breslow index 2.5 mm. Staging showed evidence of lymph nodal metastases and the patient was defined as pT3bN2M0 (stage IIIB). For melanoma 35, corresponding to a large lesion excised from the iliac fossa of a 66 year-old female patient (Breslow index 8.0 mm; pT4bN0M0, stage IIB), due to visible differences observed in the tumor mass during the macroscopical definition, sample was directly cut into two pieces of neoplastic tissue. Thus, the derived melanoma cell cultures Mel35-P (proliferative) and Mel35-I (invasive) were isolated and grown separately from the beginning. Although the degree of phenotypical differences appeared less pronounced than those observed in melanoma 29, also melanoma 35 displayed one cell population with an elongated bipolar shape and one more flattened epithelioid-like and less pigmented (Figure 1D-1F). Sequence analysis of the hot-spot regions in exon 11 and 15 of the BRAF gene showed wild type sequence, whereas NRAS exon 2 showed Q61R mutation in both cell populations. Cells from melanoma 8, Mel8-P (proliferative) and Mel8-I (invasive) were separated after few passages of *in vitro* proliferation based on the ability of these cells to grow both as adherent and as floating cells in a mixed condition. We also observed that the cells cultivated in suspension were able to grow either as single cell as small or extensive clusters. Additionally, both populations could spontaneously switch one into the other and vice versa (data not shown). Consequently, to maintain the free-floating culture as a stable condition, clusters of melanoma cell isolated from the medium were grown on uncoated plastic plates. Adherent cells appeared homogeneously small, bi- and pluripolar, whereas the floating population looked rounded in shape and less pigmented. Floating cells appeared to be arranged as very dynamic clusters rather than organized in compact spheres as usually reported for cancer stem cells (Figure 1G-1H). Mutation analysis of BRAF gene detected wild type sequence for hotspot regions in exon 11 and exon 15 and NRAS Q61K substitution in exon 2. The corresponding biopsy, derived from the back of the neck of a 52 year-old man presenting lymph nodal metastases presented a Breslow index of 1.6 mm and was classified as pT2bN2M1a, stage IV.

### Analysis of Wnt pathway activation in melanoma cells

**Figure 2:**
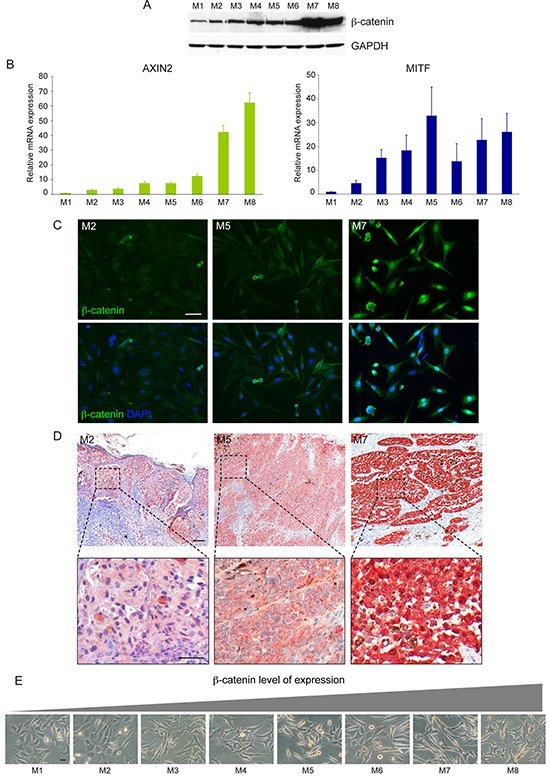
Analysis of Wnt/β-catenin signalling activation in freshly isolated low passage melanoma cell lines Immunoblot analysis of β-catenin expression in a panel of melanoma cell lines **A.** and corresponding activation of its target genes *AXIN2* and *MITF* **B.** The relative mRNA levels (± SD) of AXIN2 and MITF represent the amount of mRNA expression normalized to GAPDH mRNA expression. Total RNA was extracted from each melanoma cells at three different culture passages and was used as three independent experiments. Results were then combined for data analysis. Comparison of *in vitro* **C.** and *ex-vivo* **D.** level of β-catenin protein expression, evaluated by immunofluorescence and immunohistochemistry respectively. Phase contrast microscopic documentation of the morphological characteristics of the melanoma cell lines evaluated for β-catenin expression **E.** Scale bar: C: 50 μm; D: 100 μm and 50 μm for the higher magnification of the boxed areas; E: 20 μm.

**Figure 3:**
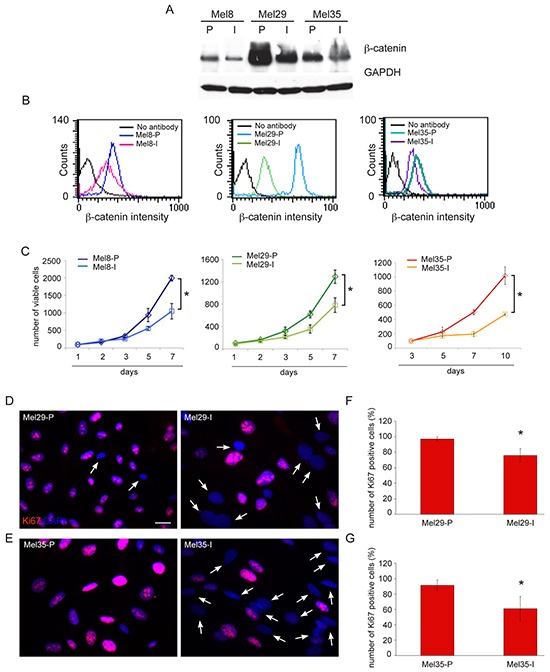
Relative higher level of β-catenin protein is associated to increased proliferative rate of melanoma cells Immunoblot **A.** and flow cytofluorimetric analysis **B.** of β-catenin level of expression in melanoma subpopulations isolated from the same biopsy. Quantitative evaluation of β-catenin level of expression in single cells, obtained by measuring FITC-β-catenin median fluorescence intensity (MFI), confirmed the homogeneous level of β-catenin protein in each cell lines. The diagram depicts a representative staining of cell line pairs. Cell growth evaluated by trypan blue exclusion assay at various time points **C.** Data are representative of three independent experiments. *p≤0.05 at the end time point. Immunofluorescence with anti-Ki67 antibody of Mel29 and Mel35 cell pairs **D, E.** Arrows point at cells detected by the nuclear 4′-6′-diamidino-2-phenyllindole (DAPI) staining, which are negative for Ki67. Scale bar: 20 μm. Percentage of Ki67-positive cells evaluated counting for each melanoma cell populations, a total of 500 cells observed in 10 fields **F, G.** Results are expressed as mean values ± SD. **p≤0.01.

Firstly, we evaluated the level of expression of β-catenin in a panel of freshly isolated melanoma cells by western blot analysis. Quantitative analysis of β-catenin protein as well as its functional activation, measured by quantification of mRNA levels of the ubiquitously target gene AXIN2 and of the melanocyte specific marker MITF showed a heterogeneous level of WNT pathway activation in melanoma cells (Figure 2A, 2B). Cell lines expressing large amount of β-catenin showed nuclear localization of the protein associated with high level of β-catenin-dependent transcriptional activity (Figure 2B, 2C). A positive correlation was found between the densitometric results of β-catenin expression obtained by western blot analysis and the mRNA levels for AXIN2 (r=0.82) and MITF (r=0.61). The comparison of β-catenin immunohistochemical staining of original tumor tissue with the corresponding melanoma cell lines confirmed the reliability of patient-derived cells isolated *in vitro* (Figure 2D). Light microscopic examination revealed an interesting relationship between β-catenin level of expression and cell morphology. In general, we observed that high level of β-catenin corresponded to high dendritic and elongated shape. By contrast, epithelioid-like enlarged cells showing de-differentiated morphology were associated with low level of β-catenin expression and poor activation of downstream target genes (Figure 2E). The association between the different β-catenin level of expression and morphological characteristics was also confirmed in tumor samples presenting two distinct cell populations (Figure 3A) suggesting that the intra-tumor heterogeneity found in these samples reflects biological differences of tumor cells linked to the status of WNT pathway activation. Cytofluorimetric analysis showed an intra-sample homogeneous staining intensity in each cell population confirming the uniformity in terms of β-catenin protein level of expression (Figure 3B). DNA sequence analysis of the β-catenin gene revealed in Mel29-P cells a point mutation into codon 44 (CCT→CTT) neighboring an important GSK3β phosphorylation site (Ser45) (Supplementary Figure 2A). The altered targeting to the proteasomal degradation could explain the consistent stabilization of the corresponding protein. All the other cell lines analyzed, including Mel29-I (Supplementary Figure 2B), showed wild-type β-catenin gene sequence, demonstrating that Mel29 cells represent two distinct tumor clones. Since cells isolated from the same tumor lesion with a clinically defined disease stage and an homogeneous genetic background, but presenting hyper or hypoactivated WNT pathway represent a realistic model to study the contribution of WNT signaling on melanoma biology, we focused the study on these cells (Mel29, Mel8 and Mel35). The proliferation rate evaluated by Trypan blue exclusion assay at various time points showed the ability of one of the two cell populations to grow significantly faster than its counterpart. For melanoma 29, one population displayed a higher degree of growth, evident from day 3, with a 0.5 and a 2-fold increase respect to the other cell population at day 3 and at days 5 and 7, respectively. Similar behavior was observed for adherent cell population isolated from melanoma 8 compared to its more slowly proliferating floating counterpart. Considering the overall slow proliferation rate of melanoma 35, growth level was measured at longer time points (5, 7 and 10 days). The results revealed an approximately 1, 1.5 and 3-fold increase in the growth of Mel35-P respect to Mel35-I at time 5, 7 and 10, respectively (Figure 3C). Quantitative immunofluorescence analysis using an anti-Ki67 antibody to identify cycling cells confirmed the presence of a lower percentage of melanoma cells displaying Ki67 positivity in the slow-growing cell populations (Figure 3D-3G).

### Heterogeneity in the migratory behavior of the freshly cultured melanoma cells: invasive versus proliferative phenotype

**Figure 4:**
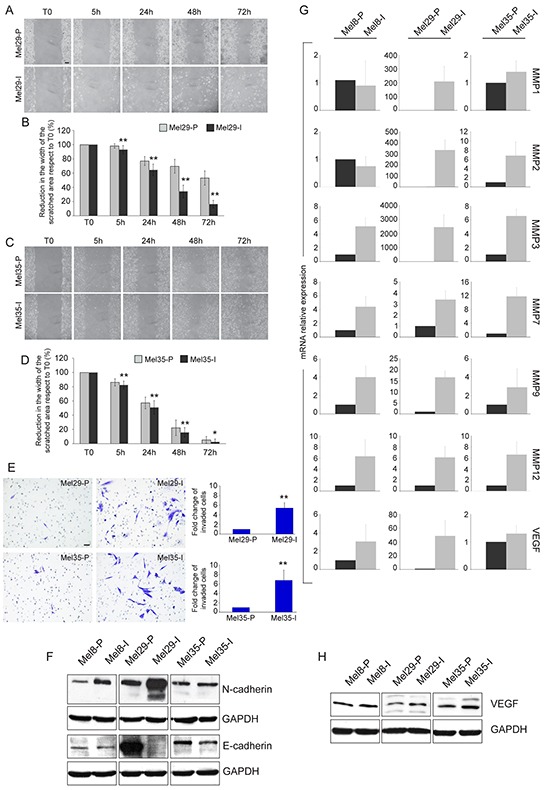
Migration and invasion assays and expression of invasive markers Cellular migration toward the scratched area was evaluated after creating a standardized cell-free area with a plastic pipette tip. Cells were allowed to grow for 0, 5, 24, 48 and 72 hours and then photographed under a microscope **A, C.** Images are representative of three separate experiments. Quantitative analysis of wound-induced migration **B, D.** Data are presented as means ± SD of three independent experiments. *p <0.05, **p≤0.01. Matrigel-invasion assay and count of the invaded cell number evaluated at 48 hours. Results are expressed as fold change of invasive cell subpopulation respect to the proliferative one, which was set as 1 for definition **E.** Data are presented as means ± SD of two independent experiments. **p≤0.01. Immunoblot analysis of N- and E-cadherin expression in Mel8, Mel29 and Mel35 cell line pairs **F.** Gene expression analysis of a panel of MMPs and VEGF normalized to GAPDH mRNA expression **G.** For each patient total RNA was extracted from cells cultures at three different passages. Values represent the means ± SD of fold-increase considering arbitrarily the proliferative subpopulation as control (value=1). Immunoblot analysis of VEGF expression **H.** Scale bar A, C, E: 50 μm.

**Figure 5:**
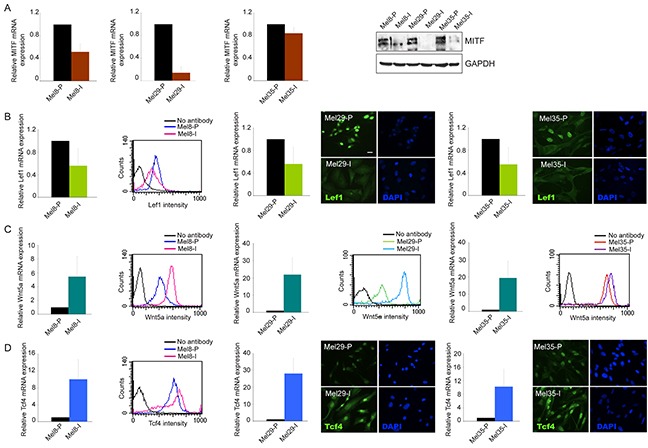
Analysis of Wnt/β-catenin pathway-associated markers Analysis of MITF **A.** LEF1 **B.** WNT5A **C.** and TCF4 **D.** at mRNA and protein level of expression in Mel8, 29 and 35 cell pairs. For mRNA quantification total RNA was extracted from cell cultures at three different passages. Values represent the means ± SD of fold-increase considering arbitrarily the proliferative subpopulation as control (value=1). LEF1 and TCF4 protein detection was performed by cytofluorimetric analysis (Mel8) or by immunofluorescence (Mel29 and Mel35). Immunoblots and cytofluorimetric analyses are representative of several independent experiments. Scale bars: 20 μm.

Tumor progression is driven by the combination of increased growth rate and invasiveness. To evaluate whether the two melanoma cell populations identified in each tumors displayed a different migratory and invasive potential, we performed the wound scratch and the matrigel invasion assays on cells isolated from melanoma 29 and melanoma 35. Cells obtained from melanoma 8 were not evaluated because of their peculiar feature to growth in suspension. Cellular migration toward the scratched area was evaluated at 5, 24, 48 and 72 hours. A larger covered area, measured as the reduction of the distance between the scratch edges compared to T0, was observed for the invasive cell cultures isolated from both the tumors at all time points tested (Figure 4A-4D). The same subpopulation displayed a parallel increase in the number of invaded cells into Matrigel at 48h, with about a 5.5-6 fold higher invasion rate respect to their proliferative counterpart, indicating a higher migrating/invasive ability of one cell culture respect to the other (Figure 4E). In all the cases the migratory and the invasive potentials showed anti-correlation with the proliferation rate supporting the biphasic model proposed for melanoma progression wherein melanoma cells exist in two states of proliferative and invasive phenotypes [38]. The expression of N- and E-cadherins, two adhesion molecules known to be respectively increased and decreased during melanoma invasion [42–43], were also analyzed. Cell cultures with higher mobility grade expressed N-cadherin as prevalent adhesion protein and low (Mel8-I and Mel35-I) or absent (Mel29-I) E-cadherin confirming the invasive pretension (Figure 4F). According with the concept that invasive growth of tumors depends on remodeling of stromal architecture, the expression of matrix metalloproteinases especially MMP3, MMP7, MMP9 and MMP12 was higher with different extend in Mel8-I, Mel29-I and Mel35-I cells compared to their proliferative counterpart (Figure 4G). We also observed increased levels of MMP2 in Mel29-I and Mel35-I, whereas MMP1 gene expression was augmented only in Mel29-I. Moreover, the production of the angiogenic molecule vascular endothelial growth factor (VEGF), a well-known marker associated with melanoma metastatic process [44], was up-regulated in invasive cell cultures at mRNA and protein levels (Figure 4G, 4H). According with previous studies [38–39], we observed a higher expression level of MITF and LEF1 in proliferative phenotype cells *in vitro* (Figure 5A, 5B). Correspondingly, the expression of WNT5a and TCF4 was higher in invasive phenotype cells (Figure 5C, 5D). Thus, based on signature profile the cells pairs segregated into distinct expression clusters previously implicated in the epithelial-to-mesenchymal-like transition [38]. However, in contrast with precedent reports [39] we observed increased level of β-catenin expression and higher nuclear distribution in proliferative cells, as assessed by both immunofluorescence and western blot analyses (Figure 3 and Supplementary Figure 3). Accordingly, the expression of *AXIN2*, a WNT downstream gene implicated in the feedback regulation of WNT/β-catenin signal [45–46], was significantly higher in the cell cultures presenting abundant nuclear β-catenin (Table 1). The general evaluation of β-catenin-dependent transcriptional activity by luciferase reporter gene expression (TopFlash/FopFlash plasmids) showed significant differences only in the case of melanoma 29, whereas cell populations isolated from patient 8 and patient 35 displayed minimal differences (data not shown), indicating that the activity of synthetic multimerized LEF/TCF binding site upstream to the Thymidine Kinase (TK) minimal promoter may discriminate only marked differences in β-catenin protein abundance. The overall analysis of a wide panel of β-catenin target genes revealed a more complicated scenario with relative high level of WNT5a, VEGF, TCF4, fibronectin, WISP1, SOX9, p16 and MMPs mRNAs and relative low level of LEF1, MITF, cyclinD1, c-MYC and E-cadherin mRNAs in the low proliferative/high invasive populations (Mel29-I, Mel8-I and Mel35-I) and the opposite expression profile in the high proliferative/low invasive populations (Mel29-P, Mel8-P and Mel35-P). Results regarding all the β-catenin target genes are summarized in Table 1.

**Table 1:** Analysis of β-catenin target genes expression in melanoma cell pairs

mRNA	Mel8-P	Mel8-I	Mel29-P	Mel29-I	Mel35-P	Mel35-I
**AXIN2**	1.0	0.52±0.22	1.0	0.06±0.05	1.0	0.59±0.26
**BCL2**	1.0	0.29±0.09	1.0	0.09±0.01	1.0	0.66±0.12
**CyclinD1**	1.0	0.92±0.23	1.0	0.45±0.24	1.0	0.53±0.25
**c-MYC**	1.0	0.45±0.21	1.0	0.50±0.35	1.0	0.80±0.35
**CD44**	1.0	1.0±0.32	1.0	4.62±3.10	1.0	1.24±0.56
**DKK1**	1.0	0.42±0.35	1.0	22.2±9.80	1.0	1.93±0.80
**E-cadherin**	1.0	1.2±0.30	1.0	0.02±0.05	1.0	0.58±0.20
**Fibronectin**	1.0	3.27±1.80	1.0	4.08±2.16	1.0	1.57±0.90
**IL-6**	1.0	0.51±0.07	1.0	1049.0±391	1.0	0.74±0.40
**IL-8**	1.0	6.88±2.90	1.0	364.8±65.5	1.0	1.30±0.32
**LEF1**	1.0	0.56±0.20	1.0	0.12±0.11	1.0	0.55±0.32
**MITF**	1.0	0.51±0.14	1.0	0.14±0.10	1.0	0.84±0.11
**MMP2**	1.0	0.80±0.30	1.0	334.0±72.0	1.0	6.78±3.30
**MMP7**	1.0	4.35±1.60	1.0	3.35±2.60	1.0	11.8±2.60
**MMP9**	1.0	4.02±1.10	1.0	16.6±4.20	1.0	2.80±1.65
**PAX3**	1.0	0.53±0.16	1.0	0.28±0.13	1.0	1.32±0.53
**p16**	1.0	2.00±0.47	1.0	2.17±0.68	1.0	1.78±0.10
**SOX2**	1.0	0.36±0.14	1.0	0.55±0.30	1.0	2.70±2.00
**SOX9**	1.0	9.20±3.30	1.0	3.12±0.63	1.0	28.5±12.7
**Survivin**	1.0	0.54±0.41	1.0	1.20±0.22	1.0	0.98±0.55
**WIF**	1.0	8.30±4.40	1.0	0.11±0.09	1.0	42.0±13.4
**WISP1**	1.0	12.0±9.40	1.0	12.2±3.60	1.0	11.4±8.67
**WNT5A**	1.0	5.45±2.90	1.0	21.0±9.60	1.0	19.4±9.70
**VEGF**	1.0	4.04±1.80	1.0	46.6±14.0	1.0	1.23±0.38
**TCF4**	1.0	10.0±4.72	1.0	28.1±9.0	1.0	10.2±5.16

Values indicating increases and decreases were reported in red and blue characters, respectively

Mel8, that shifts back and forward between the proliferative and the invasive phenotype in a very dynamic fashion, gave us some additional important information. Adherent confluent Mel8 cells express higher level of WNT5a, TCF4 and VEGF and lower level of LEF1 at mRNA level than sub-confluent cells (Supplementary Figure 4A) suggesting that these cells are prone to phenotype switch, simulating *in vitro* tumor spreading. Since change of WNT5a expression is an early event observed in association with the increased cell density, one possible explanation is that the autocrine secretion of WNT5a, a regulator of β-catenin protein [47], could in some cases prime the phenotype switching. Supporting this hypothesis, cytofluorimetric analysis revealed that in over-confluent adherent Mel8 cells, low and high-WNT5a expressing cells coexisted as discrete subpopulations. In this case the level of WNT5a protein reached, in a portion of cells, a level similar to that of the floating cells (Supplementary Figure 4B). Moreover, treatment of adherent Mel8-P cell with recombinant full length Wnt5a (rWnt5a) increased the expression of TCF4, mimicking the effects of the autocrine factor (Supplementary Figure 4C). These data suggest that some melanomas gain an intrinsic metastatic potential independent to extrinsic effectors such as the environmental context of primary melanoma. In fact, in the case of Mel8 cell heterogeneity persisted with a steady-state stable equilibrium also in *in vitro* growth, in absence of specific selective pressure imposition.

### Cellular phenotype heterogeneity in melanoma tissue samples

**Figure 6:**
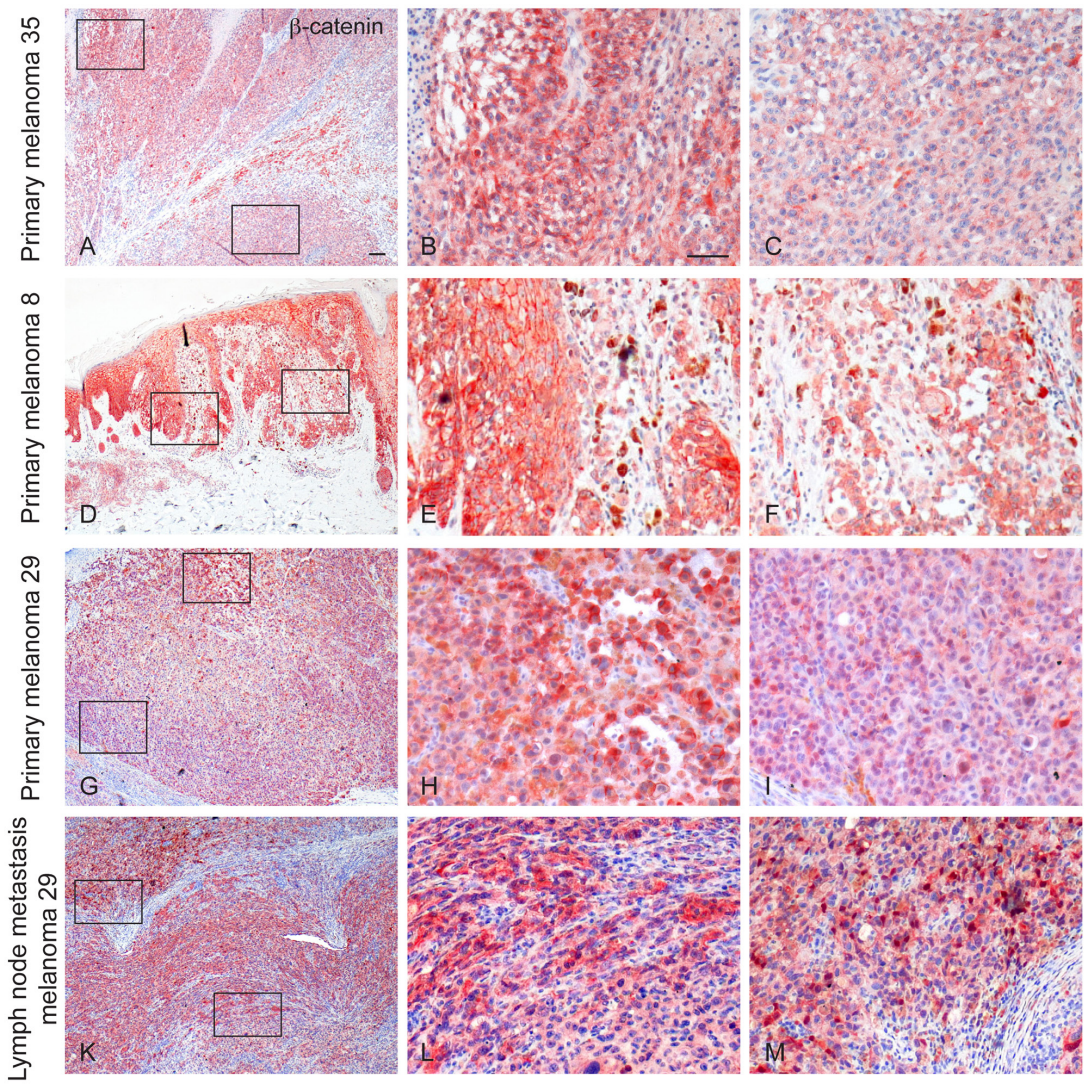
Immunohistochemical expression of β-catenin in primary and metastatic melanomas An heterogeneous staining pattern for β-catenin both in intensity and localization is evident among the different tumour samples and within the same specimen. Examples of β-catenin immunoreactivity in primary melanomas (Mel35 in **A.** and enlarged view of the boxed areas in **B.** and **C.** Mel8 in **D.** and enlarged view of the boxed areas in **E.** and **F.** Mel29 in **G.** and enlarged view of the boxed areas in **H.** and **I.** and in lymphnode metastases (Mel 29 in **K.** and enlarged view of the boxed areas in **L.** and **M.**) are shown. Scale bar: A, D, G, K: 100 μm; B, C, E, F, H, I, L, M: 50 μm.

We next investigated whether the marked phenotypical differences identified in the three freshly isolated melanoma cells pairs could be artificially driven by the *in vitro* culture system or were naturally generated during tumor evolution process. To this aim, we evaluated by immunohistochemistry the expression of β-catenin in the corresponding formalin-fixed, paraffin-embedded melanoma samples. The tumor specimens appeared positively stained for β-catenin, although the staining pattern resulted heterogeneous both in intensity and localization. The heterogeneous intensity was not randomly distributed. In fact, cells expressing high (or low) level of β-catenin were localized in different areas defining distinct tumor regions. In melanoma 35, the immunostaining appeared mainly localized in the cytoplasm of the tumour cells with few scattered areas showing also a membranous reactivity (Figure 6A-6C). Melanoma 8 displayed a membranous and cytoplasmic reactivity in all the tumour cells but with a heterogeneous level of intensity. We also observed single cells and small groups of cells showing intense staining in the nuclear compartment (Figure 6D-6F). In melanoma 29, β-catenin staining was found for the most part in the cytoplasm, with some tumor cells presenting also plasma membrane positivity. Moreover, cells displaying nuclear staining were clearly detected in some localized tumor areas scattered throughout the entire lesion (Figure 6G-6I). Two lymph node metastases from the latter patient were also evaluated and showed a discontinuous level of immunoreactivity for β-catenin similar to that observed in the primary lesion, with cytoplasmic, membranous and nuclear staining patterns (Figure 6K-6M). These data support the idea that different populations co-existed in the original biopsies and were not originated from the experimental procedures used for the setting-up and maintenance of the cell cultures. Since in melanoma 29 the two populations were also characterized for the different genetic signature for β-catenin gene, we used laser tissue microdissection technique to isolate small groups of cells presenting high or low level of β-catenin expression and to perform sequence analysis. In both primary and metastatic samples, cells isolated from areas characterized by an intense β-catenin expression, presented the mutation in its gene, whereas the sequence mutation was absent in the DNA derived from cells with low level of β-catenin (Supplementary Figure 5). These results unequivocally demonstrated that the two clones originally co-existed in different regions of the tumor sample. Additionally, the immunohistochemical analysis of MITF expression on serial sections of the same samples revealed higher immunolabelling for this melanocyte differentiation marker mainly on areas characterized by a strong reactivity for β-catenin, further confirming that the phenotypical features highlighted *in vitro* match with the original tumour (Supplementary Figure 6).

**Figure 7:**
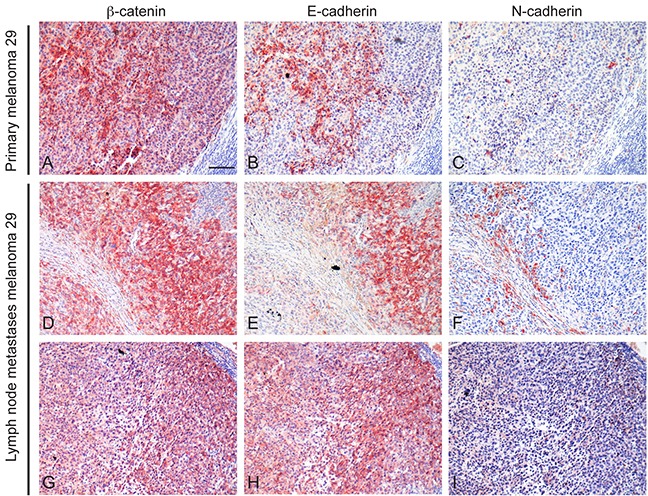
Immunohistochemical analysis of β-catenin, E-cadherin and N-cadherin expression Serial sections of paraffin-embedded primary and lymph nodal metastases of patient 29 were analyzed for β-catenin **A, D, G.** E-cadherin **B, E, H.** and N-cadherin **C, F, I.** expression. Positive E-cadherin immunolabelling was mainly localized in tumour areas presenting high β-catenin reactivity whereas N-cadherin staining was weak and predominantly observed in tumour cells presenting feeble staining for β-catenin. Scale bar: 100 μm.

In addition, analysis on tissue paraffin-embedded on both the primary melanoma 29 and the corresponding lymph node metastases showed positive reactivity for E-cadherin primarily in tumour areas presenting high β-catenin reactivity. In general, N-cadherin staining was weak and/or undetectable both in the primary lesion and in the metastases. However, a high immunoreactivity was predominantly observed in tumour cells presenting a more atypical morphology and feeble staining for β-catenin and E-cadherin (Figure 7).

### Comparison of the effect of chemotherapeutic agent cisplatin on proliferative and invasive melanoma cells

**Figure 8:**
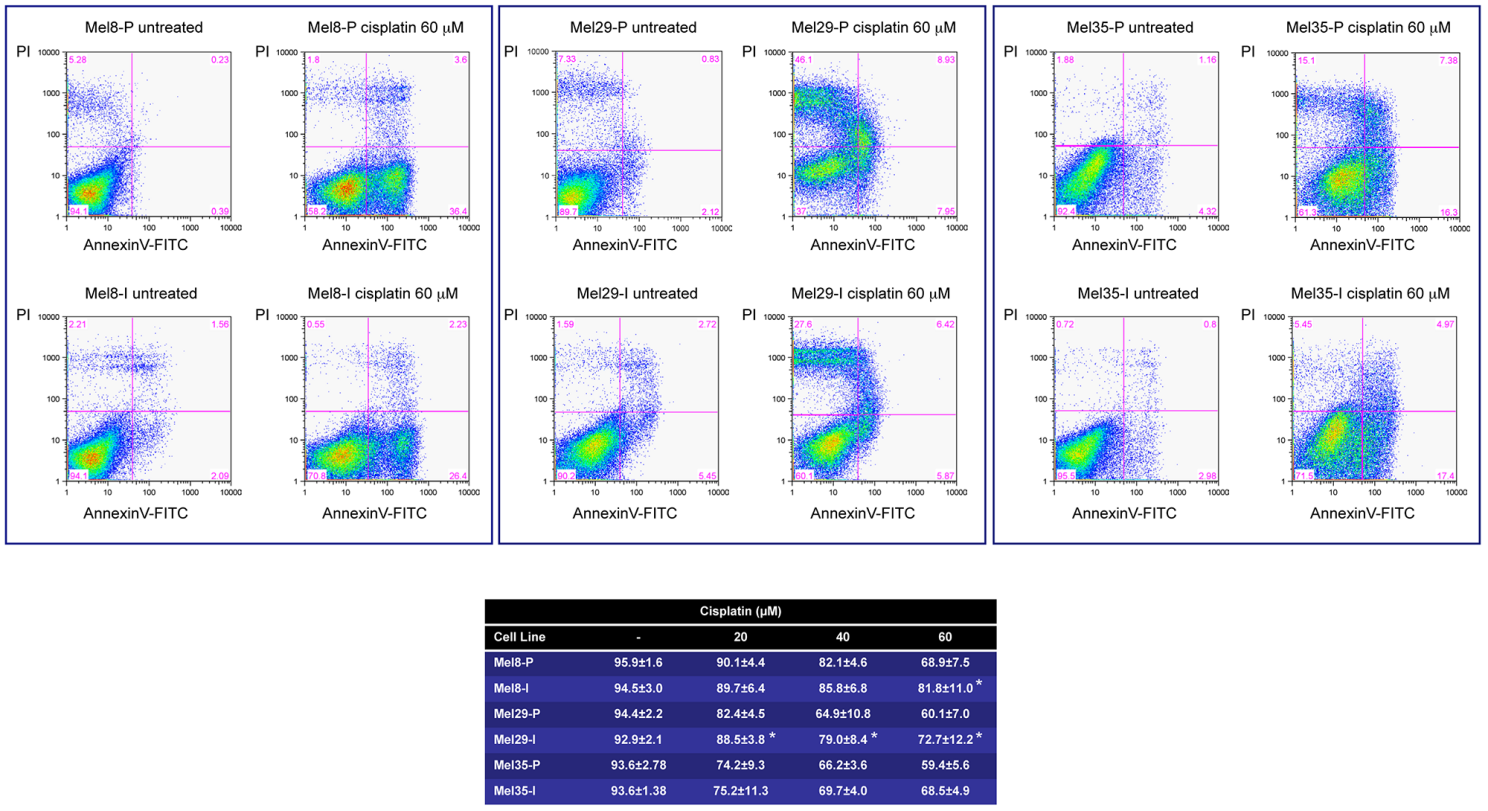
*In vitro* sensitivity of melanoma cells to cisplatin Mel8, 29 and 35 cell pairs were cultured in the presence of different doses (20-40-60 μM) of cisplatin for 24 hours. Death and apoptosis were evaluated by FACS analysis using annexin V/iodide propidium staining. Dot plots show one representative experiment at the highest dose. For all the doses tested the number of surviving cells (Annexin V -/PI -) are reported in the Table. Data presented are the mean ± SD of three independent experiments. *p <0.05.

We next investigated the cell susceptibility to chemotherapy-induced apoptosis consisting of cisplatin treatment. In dose-dependent experiments both cell phenotypes underwent cell death. However, invasive cells demonstrated resistance to cisplatin treatment compared to proliferative phenotype especially at the higher doses (Figure 8). Even if multiple mechanisms may contribute to cisplatin resistance, the demonstration that WNT/β-catenin pathway is implicated in facilitating melanoma cells apoptosis [48] suggests that, at least in part, reduced β-catenin level of expression contributes to invasive phenotype chemoresistance. It is interesting to note that the expression of the anti-apoptotic protein BCL-2 was unexpectedly decreased in Mel8-I and Mel29-I resistant cells, suggesting that BCL-2 enhances rather than reduces chemosensitivity in melanoma cells (see Table 1). In line with our results, the co-overexpression of BCL-2 and β-catenin has been reported in other tumors [49] and the transcriptional regulation of BCL-2 by MITF has been documented in primary melanocytes and melanoma cells [50]. Thus, our data demonstrated that different subpopulations, coexisting in the same tumor, could have different response to therapy based on specific gene expression profile signature.

### The phenotype signature influences melanoma cell pro-inflammatory state

**Figure 9:**
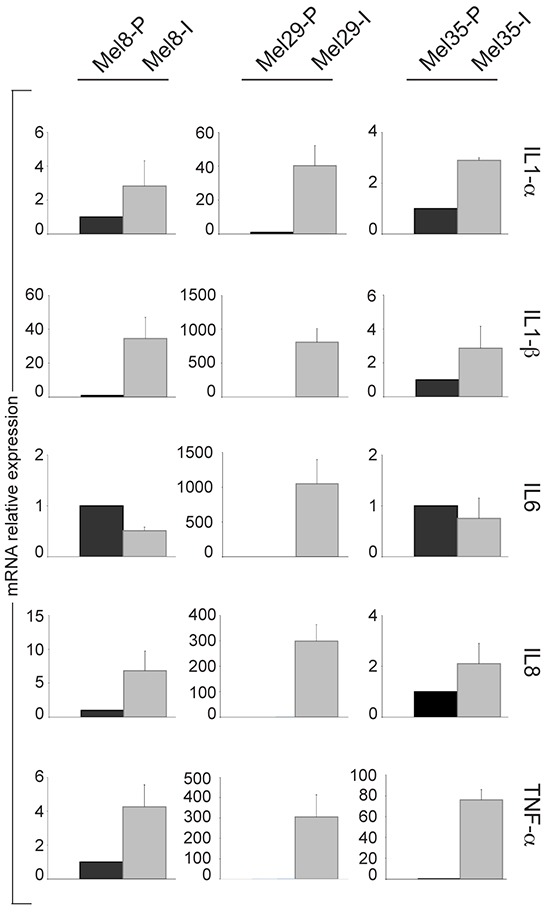
Analysis of the expression of TNF-α inducible genes in melanoma cell line subpopulations Analysis of IL1-α IL-1β, IL6 and IL8 and TNF-αmRNA across melanoma cell lines pairs 8, 29 and 35. Total RNA was extracted from cell cultures at three different passages. Values represent the means ± SD of fold-increase considering arbitrarily the proliferative subpopulation as control (value=1).

In the past years, inflammatory signals emerged as a crucial promoter of phenotypic plasticity in melanoma [51]. Studies focused on the reciprocal interactions of melanoma and immune cells demonstrated that microenvironment-derived tumour necrosis factor (TNF)-α might induce dedifferentiation of the melanoma cells [52]. Thus, we studied the pro-inflammatory pathway activity by measuring the expression of TNF-α inducible genes IL1-α, IL1-β, IL6 and IL8 across melanoma cell lines pairs. Among the cytokines tested, IL1-α, IL1-β and IL8 were noticeably increased in all the cells with the invasive phenotype whereas IL6 resulted up-regulated only in Mel29-I (Figure 9). Moreover, autocrine production of TNF-α was augmented in Mel8-I, Mel29-I and Mel35-I compared to their proliferative counterpart cell line suggesting that, in addition to the possible contribution of tumour microenvironment immune cells, invasive melanoma cells autonomously facilitate metastatic spread supporting local immunosuppression.

## DISCUSSION

Tumor progression is driven by molecular changes conferring a proliferative advantage and promoting invasive and metastatic phenotypes. Deregulation or mutations of WNT/β-catenin pathway are implicated in both tumour formation and progression of various cancer types. However, despite the fact that melanoma was one of the first tumors associated with β-catenin dysregulation [53], its role in this deadly disease remains still not fully elucidated. The major discrepancies emerged from the comparison between studies performed with melanoma cell culture model and investigations based on immunohistochemical analyses in skin biopsy and clinical outcome. *In vitro* studies proposed that an increased nuclear translocation and activity of β-catenin promote melanoma proliferation [54] and invasion [55]. On the other hand, other studies linked the activation of WNT/β-catenin signaling to a decreased proliferation [23] and to a repression of invasion [56] and migration [57]. *In vivo* observations from melanoma patients indicated that nuclear β-catenin correlates with improved survival [23; 30]. These data collectively suggest that the ambiguous final outcome, effect of β-catenin overexpression in the melanocyte lineage strongly depends on the combination of both intracellular and microenvironmental contexts. The majority of melanoma research is conducted using established cell lines maintained in culture for a long period and presenting evidence of deviations from the phenotype of the originating tumor. For example, β-catenin mutations are rare in primary melanoma specimens [58 and our unpublished data], in comparison to well-characterized melanoma cell lines cultured *in vitro* for a long period [53]. It is possible that the higher proliferative rate of β-catenin mutated cells facilitates the *in vitro* adaptation, thus promoting the selection of subclones carrying this genetic defect and therefore attenuating the natural tumour heterogeneity. In light of these considerations, we restricted data analysis on short-term cell cultures and we matched the *in vitro* results with the corresponding surgical specimens. Using this methodological approach we demonstrated the presence of two major molecular and biological melanoma phenotypes linked to the regulation of WNT/β-catenin signaling pathway. One subpopulation, carrying high level of β-catenin, displayed a proliferative and more differentiated phenotype (high Ki67, cyclinD1 and MITF). The other one, with relative low amount of β-catenin, showed a more invasive phenotype typical of the mesenchymal-like transition (high MMPs and VEGF). Changes in the gene/protein expression profile have been previously linked to the dynamic and reversible phenotypic tumor cell plasticity [38,59]. The critical role of β-catenin in the generation of diverse melanoma intra-sample subpopulations is unequivocally proven by data collected on cells isolated from patient 29. Due to the occurrence of the mutation in the exon 3 of β-catenin gene, the derived cell lines showed the strongest differences in gene expression profile, mitotic index, migration rate and morphological features. Moreover, the phenotype switching appeared most likely irreversible. By contrast, Mel8, that repeatedly transits back-and-forth between states of proliferation and invasion, demonstrated that the expression of angiogenic and prometastatic factors (VEGF, MMPs), as well as the modification of morphological and adhesion properties (e.g. cadherins) occurring during invasion, can rapidly and spontaneously reverse. Therefore, these observations raise questions on how this process is regulated also *in vitro*, in absence of microenvironmental conditioning signals. In fact, even if high cell density lightly encouraged cells detachment and the free-floating growth, the adherent/floating cells ratio is nearly constant (data not shown). One interesting observation is the enhanced expression of WNT5a, an autocrine factor implicated in β-catenin regulation, in over-confluent Mel8 adherent cells. Thus, it is possible that elevation of WNT5a represents an important step to prime cell detachment and to favour the acquisition of the metastatic phenotype. In line with this idea a modest increase of WNT5a in over-confluent melanoma cells was observed also in other cell lines (data not shown). The clonal genetic diversity of β-catenin gene and the consequent protein hyperfunction in Mel29 cells mostly underlined the dissimilarity between gene expression profile and pathways activation. However, comparable results were obtained with Mel8 and Mel35 cell pairs that displayed moderate differences in β-catenin expression, demonstrating that the relative amount of β-catenin protein, only in part, explains variations in target genes expression. In these cases the relative difference of β-catenin expression could be explained by the action of several mechanisms including the interactions with different partners or the influences of canonical and non canonical Wnt pathway factors, such as Wnt3a and Wnt5a. Since the WNT/β-catenin target genes list includes important regulators of cell proliferation as well as mediators of invasion, aberrant β-catenin over-expression alone could not satisfactorily explain the phenotypic differences. As previously proposed [38], our data suggest that LEF1 and TCF4, two direct target genes of β-catenin signaling [60], or more likely the ratio LEF1/TCF4, are important in phenotype definition. In fact, the metastatic phenotype lightly reduces the expression of LEF1 but, at the same time, strongly up-regulates the expression of TCF4. Notably, even if all LEF1/TCFs bind to the same consensus sequence, some alternatively spliced variants produced from *TCF* genes, but non from *LEF1* gene, encode a non-sequence specific DNA-binding domain and a region that facilitates p300 interaction [61–62]. These isoforms of TCFs are the most potent forms of TCFs able to regulate specific WNT target genes with lower-affinity WNT responsive elements [63]. By contrast, LEF1 activates the expression of lineage specific marker MITF, while TCF4 does not [39]. Thus, it is conceivable that the preferential expression of LEF1 or TCFs could direct β-catenin activity to different subsets of WNT target genes in melanoma cells. According with the idea that LEF1/TCFs activities create inside the nucleus complex signals regulating β-catenin activity that does not simply correlate to the level of β-catenin protein with an ON/OFF state, we observed that the use of artificial WNT reporter plasmid (i.e. TopFlash) does not reflect slight variation of β-catenin protein amount. Gene expression analysis of WNT signaling target genes evidenced two distinct signatures in all the three cells pairs with some interesting exceptions. For example, the down-regulation of PAX3 and SOX2, two transcription factors important for melanocytic proliferation, migration and differentiation [64–65], was not confirmed in Mel35. Another interesting point is the opposite regulation of WIF1 and IL6 (respectively down and up-regulated in the invasive subpopulation) in Mel29 in comparison to Mel8 and Mel35. An intriguing observation is the strong increase of WISP-1 gene expression in the invasive subpopulations since a higher expression of WISP-1 has been linked to pulmonary metastasis [66] and to local suppression of immune response in the B16 melanoma model.

One of the most interesting open questions is the clinical relevance of the coexistence of cells presenting different gene expression signature in melanoma progression. Results presented in this manuscript demonstrated that cells displaying epithelial-to-mesenchymal transition could also play a role in resistance to therapy. Thus, it is possible that the different proportions of each melanoma subpopulation could be modified in response to chemotherapy, resulting in the expansion of the migratory cell fraction and in the promotion of melanoma metastasis. These observations were reinforced by examining the activity of the inflammatory pathway. Interestingly, melanoma cell lines greatly differed in the production of inflammatory signals, suggesting that melanoma phenotypes actively influence the immune cell composition of the tumor microenvironment with important implications also for melanoma immune response and immunotherapeutic strategies. Consistent with our results previous studies showed that dedifferentiated melanomas are associated to an extensive infiltration of myeloid immune cells [52]. Very recently, an inverse relationship of active β-catenin signaling and T cell infiltration in both human melanoma samples and transgenic melanoma mouse models has been reported [67], reinforcing the link between the WNT pathway and immune resistance.

In conclusion, our data demonstrate that in melanoma the tumor-intrinsic heterogeneity of WNT signaling regulation is associated with specific marker signatures and reflects the biological complexity of the disease, thus contributing to explain phenomena such as tumor growth, progression, self-renewal, and capacity to therapy resistance.

## MATERIALS AND METHODS

### Melanoma cell cultures

Melanoma specimens were obtained from patients enrolled by the Melanoma Unit of the San Gallicano Dermatologic Institute, Istituti Fisioterapici Ospitalieri (IFO). Melanoma cells were isolated exclusively from excess parts of the biopsy collected for histological examinations without compromise the standard diagnostic procedure. Institutional Research Ethics Committee (IFO), approval was obtained to collect samples of human material for research. The Declaration of Helsinki Principles was followed and patients gave written informed consent. The tissue was manually crumbled in small pieces and then incubated with collagenase 0.35% for 45 minutes at 37°C, centrifuged, resuspended and grown in OptiMEM (Life Technologies, Invitrogen, Milan, Italy) medium containing 10% fetal bovine serum and antibiotics. All the experiments were performed at low cell culture passages (2–12).

### Melanin content determination

Extracellular melanin release was measured as previously described [68]. Briefly, 200 μl of the media was removed and the absorbance was measured spectrophotometrically at 405 nm using a plate reader to measure extracellular melanin. After extraction of the protein fraction, cell pellets were dissolved in 200 μl of 1 M NaOH for 2h at 60°C and the absorbance was measured as above. Standard curves using synthetic melanin (0-250 mg/ml) were prepared for each experiment. Melanin production was calculated by normalizing the total melanin values with protein content (μg melanin/mg protein).

### Cell proliferation

Melanoma cells were seeded on 35 μm plates and allowed to grow for the indicated time points and then harvested by incubation in 0.5% trypsin, 0.2% ethylenediamine tetraacetic acid (EDTA) at 37°C. Cell viability was measured by Trypan blue exclusion assay. All experiments were performed three times. To maintain the free-floating condition of Mel8-I population cells were grown on uncoated plastic plates.

### Scratch assay

Melanoma cells were seeded on 35 mm plates and allowed to grow until confluence. Cell monolayer was then scratched to create a standardized cell-free area using a 200 ml pipette tip, as previously described [69]. After repeated washes, cells were then maintained in culture and observed at different time points (immediately after the scratch (T0) and at 5, 24, 48 and 72 hours). Images were recorded using an Axiovert 25 inverted microscope (Carl Zeiss, Oberkochen, Germany) and a Power Shot G5 digital camera (Canon, Inc., Tokyo, Japan). Migration was quantified by measuring the recovered scratch area at selected time points and evaluated as percentage of reduction respect to T0.

### Invasion assay

Cell invasion was assessed using the Biocoat matrigel invasion chambers with 8 micron pores (BD, BioCoat Matrigel Invasion Chambers, BD Biosciences, Franklin Lakes, NJ, USA). 2.5×10^5^ cells resuspended in OptiMEM without FBS were seeded into the matrigel coated 6-well inserts. OptiMEM plus 10% FBS was added to the lower chamber. After 48 hours, non invading cells located in the upper side of the chamber were wiped off using cotton swabs. Invaded cells were fixed in 100% methanol and stained with tolouidine blue. Invasion was measured by counting the number of cells in 10 microscopic fields (magnification 20X) and results are expressed as fold change of invasive cell subpopulation respect to proliferative one, which was set as 1 for definition.

### Immunofluorescence

Cells were fixed with 4% paraformaldehyde for 30 min at room temperature followed by 0.1% Triton X-100 to allow cell permeabilization. Cells were then incubated with the following primary antibodies: anti-Ki67 polyclonal antibody (1:300) (Abcam, Inc., Cambridge, UK), anti-LEF1 polyclonal antibody (1:300), anti-TCF4 polyclonal antibody (1:300), anti-MITF monoclonal antibody (1:300) (Santa Cruz Biotecnology), anti-Cyclin D1 monoclonal antibody (1:300) (Zymed Laoratories, Life Tecnologies, Invitrogen) for 1 hour. Primary antibodies were visualized using anti-rabbit IgG Alexa Fluor 488, anti-mouse IgG Alexa Fluor 488, anti-goat IgG Alexa Fluor 488 (Molecular Probes, Eugene, OR, USA) or anti-rabbit IgG-AlexaFluor 555 (Cell Signaling Technology, MA, USA). Nuclei were visualized with 4′,6′-diamidino-2-phenylindole (DAPI) (Sigma-Aldrich Srl, Milan, Italy). Fluorescence signals were recorded using a CCD camera (Zeiss, Oberkochen, Germany). The percentage of positive cells for Ki67 was evaluated counting for each melanoma cell populations, a total of 500 cells observed in 10 fields and expressed as mean values ± SD.

### Immunohistochemistry

Serial sections (3 μm) derived from formalin-fixed and paraffin- embedded blocks were dewaxed in xylene and rehydrated through graded ethanol to PBS. Antigen retrieval was achieved by heating sections in 10 mM citrate buffer, pH 6 before endogenous peroxidase blocking. Tissue sections were incubated with the following primary antibodies: anti-β-catenin mouse monoclonal antibody (1:300) (Zymed), anti-E-cadherin monoclonal antibody (1:500), anti-N-cadherin (1:200) and anti-MITF monoclonal antibody (1:100) (Dako, Corp., Carpinteria, CA, U.S.A). Sections were then treated with peroxidase-labelled polymer conjugated with secondary antibodies (Dako Corp.), incubated with 3-amino-9-ethylcarbazole substrate chromogen (Dako Corp.) and counterstained with haematoxylin. Negative controls were obtained by omitting the primary antibodies from the immunohistochemical procedure.

### Tissue laser microdissection and gene sequence analysis

Laser microdissection was performed using a Nikon Eclipse TE 2000-S laser capture microscope system (Nikon Instruments, Tokyo, Japan) as previously reported [70]. Briefly, 5-μm formalin-fixed tissue sections were deparaffinized and immunohistochemically stained with anti-β-catenin antibody. Intense or weak stained areas were selected, collected by laser capture microdissection, and genomic DNA was extracted using PicoPure DNA extraction kit (Arcturus Bioscience, Mountain View, CA, U.S.A.) in a total volume of 55 μl. Genomic DNA from cell cultures was extracted using Tissue Kit (Qiagen, Milan, Italy) About 3–5 ml of genomic DNA was subject to PCR in a total volume of 50 ml containing 25 ml of 2x PCR Master Mix (Promega, Madison, WI, USA) and 25 pmol of forward primer 5′-GCTGATTTGATGGAGTTGGA-3′ and reverse primer 5′-GCTACTTGTTCTTGAGTGAA-3′. DNA fragments were checked by electrophoresis in 2% agarose gel and purified using High Pure PCR Product Purification Kit (Roche Diagnostics GmbH, Mannheim, Germany) before sequence analysis.

### Western blot analysis

Cell extracts were prepared with RIPA buffer containing proteases and phosphatases inhibitors. For extracting cytoplasmic, nuclear, membrane and cytoskeletal proteins separately Compartimental Protein Extraction Kit (Merk Millipore, Darmstadt, Germany) was used according with manufacturer instruction. Proteins were separated on SDS-polyacrylamide gels, transferred to nitrocellulose membranes and then treated with the following primary antibodies: anti-β-catenin mouse monoclonal (1:1000), anti-N-cadherin rabbit polyclonal (1:1000) (Santa Cruz Biotechnology), anti-E-cadherin mouse monoclonal (1:500) (Dako Corp.), anti-VEGF rabbit polyclonal (1:200) (Abcam, Inc.) antibodies. Anti-GAPDH rabbit polyclonal antibody (1:2000) (Santa Cruz Biotechnology) was used to normalize protein content. Horseradish peroxide-conjugated goat anti-mouse or goat anti-rabbit secondary antibody complexes were detected by chemiluminescence (Santa Cruz Biotechnology).

### Semi-quantitative RT-PCR

Total RNA was extracted from each melanoma pairs at three different cell culture passages using Aurum Total mini kit (BioRad) and were used as three independent experiments. Results were then combined for data analysis. cDNA was synthesized from 1 mg of total RNA using the FirstAid kit (Fermentas, ThermoFisher Scientific, Waltham, MA, USA) and amplified in a reaction mixture containing iQSYBR Green Supermix (BioRad) and 25 pmol of forward and reverse primers using an iQ5 Light Cycler (BioRad). All samples were run in triplicate, and relative expression was determined by normalizing results to glyceraldehyde-3-phosphate dehydrogenase (GAPDH) mRNA. Sequences of primers are reported in Supplementary Table S1. For each gene, the assessment of product specificity was performed by examining PCR melt curves after qRT-PCR.

### Flow cytometry analysis

Cells were fixed and permeabilized with Cytofix/Citoperm™ (BD Bioscience, Erembodegem, Belgium) and stained with the following primary antibodies: anti-β catenin, anti-LEF1, anti-TCF4 and anti-WNT5a (Santa Cruz Biotecnology). After washing cells were incubated with secondary antibodies. After washing cells were then analyzed by flow cytometry using a FACSCalibur (Becton Dickinson, Mountain View, CA, USA). Median Fluorescence Intensity (MFI) was evaluated on a linear scale. Cell death and apoptosis were analyzed by annexin-V FITC/propidium iodide (PI) double staining method. Cells were harvested by trypsinization, resuspended in the staining buffer (10 mM HEPES/NaOH, pH 7.4, 140 mM NaCl, 2.5 mM CaCl2), stained with FITC-labeled annexin V and PI for 15 min at RT in the dark and then kept on ice until be analyzed. Data from 5×10^4^ cells were acquired from each sample and analyzed using FlowJo software.

### Statistical analysis

Student's *t*-test was used to assess statistical significance with thresholds of * p≤0.05 and ** p≤0.01. The correlation between β-catenin expression and AXIN2 or MITF mRNA was determined by the coefficient of Pearson's test (r).

## SUPPLEMENTARY FIGURES AND TABLE


