# Supplementary material for: The role of WNT/β-catenin signaling pathway in melanoma epithelial-to-mesenchymal-like switching: evidences from patients-derived cell lines
Source: Oncotarget. 2016 May 9;7(28):43295–314. doi: 10.18632/oncotarget.9232 (PMC5190024; doi:10.18632/oncotarget.9232)
Supplement: Supplementary file 1 [file oncotarget-07-43295-s001.pdf]

SUPPLEMENTARY FIGURES AND TABLE

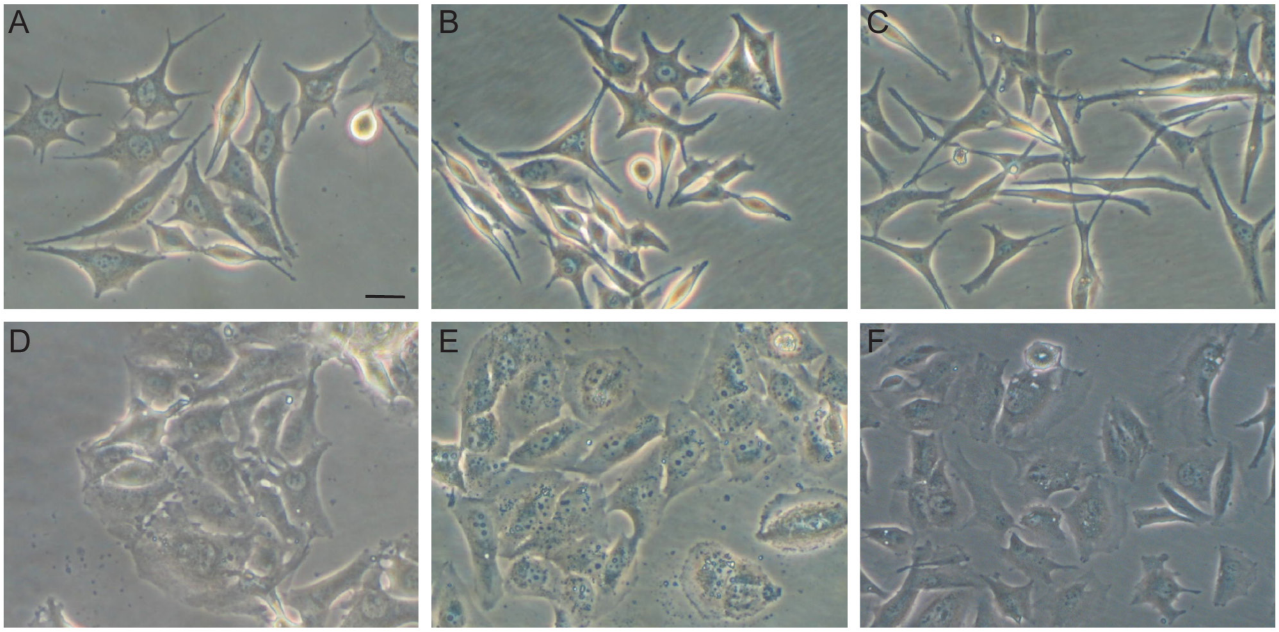

Supplementary Figure S1: In vitro morphology of low passage melanoma cell lines. Representative images of a panel of melanoma cell lines freshly isolated from different patients. Phase contrast microscopic analysis evidenced marked differences: cells morphology spanned from dendritic shape, bipolar or pluripolar, similar to that of normal human primary melanocytes **A–C.** to the flat epithelioid-like shape **D–F.** Scale bar: 20 µm.

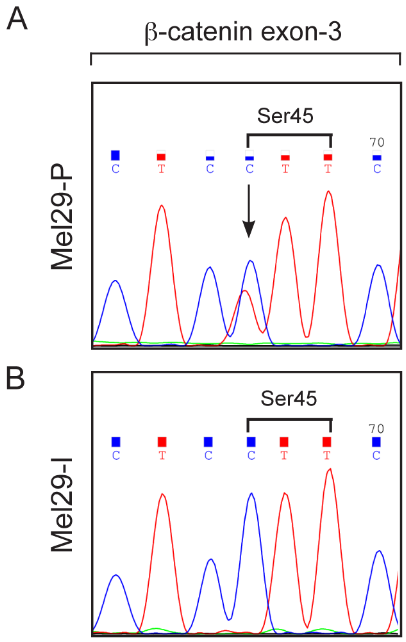

Supplementary Figure S2: Sequence analysis of β-catenin gene. DNA sequence of exon 3 of CTNNB1 gene from Mel29-P (mutated) **A.** and Mel29-I (wild-type) **B.** cell cultures.

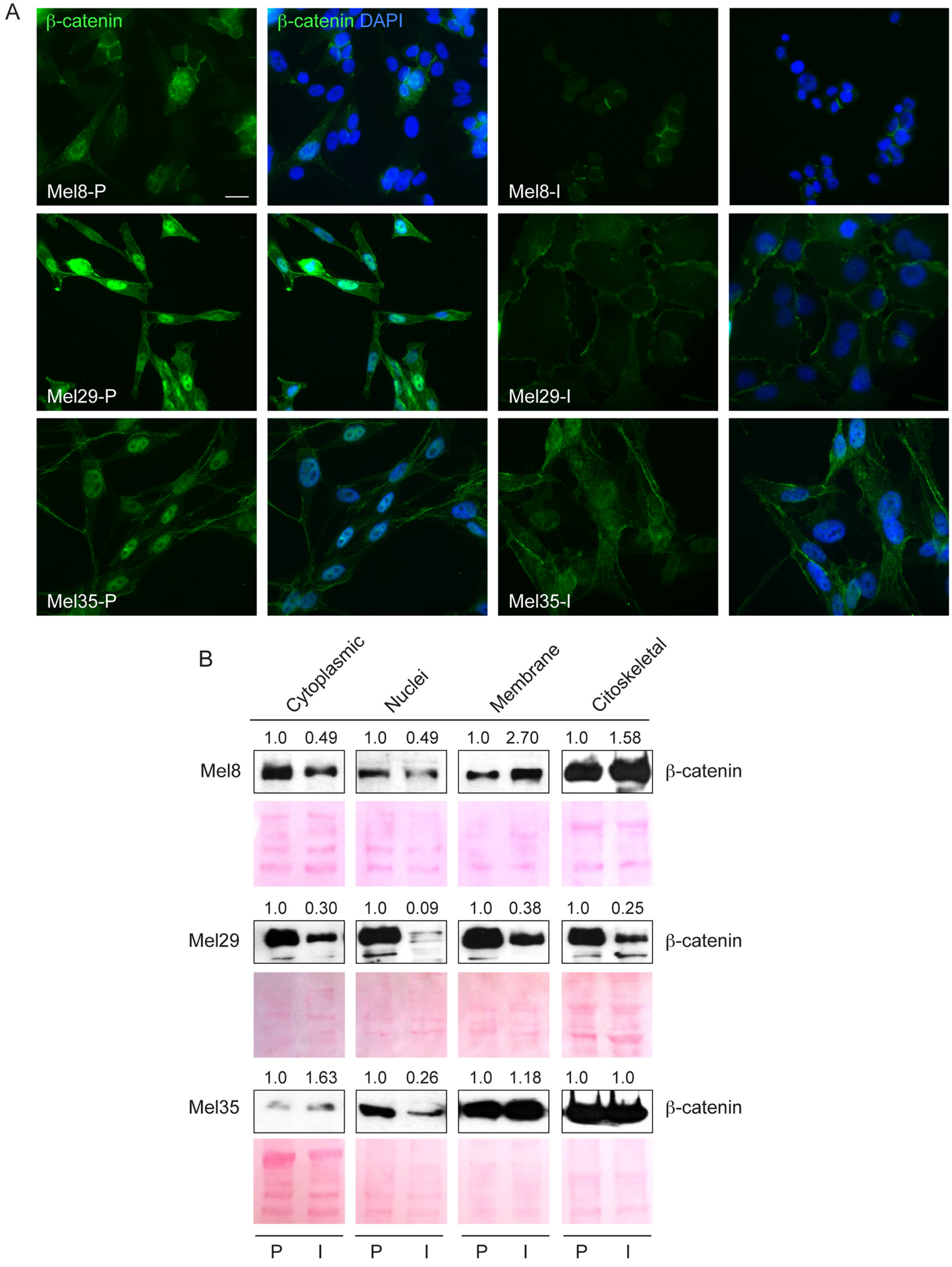

Supplementary Figure S3: β-catenin expression and localization in Mel8, Mel29 and Mel35 cell line pairs. Immunofluorescence **A.** and western blot with densitometric analysis of band intensities to quantify protein expression changes **B.** results show abundant nuclear localization in the proliferative subpopulations of the three cell line pairs in comparison to the lower signal observed in the invasive counterpart. Nuclei are counterstained with DAPI (A). Scale bar: 20 µm. Poinceau S staining of one piece of the membrane was used to check samples loading (B).

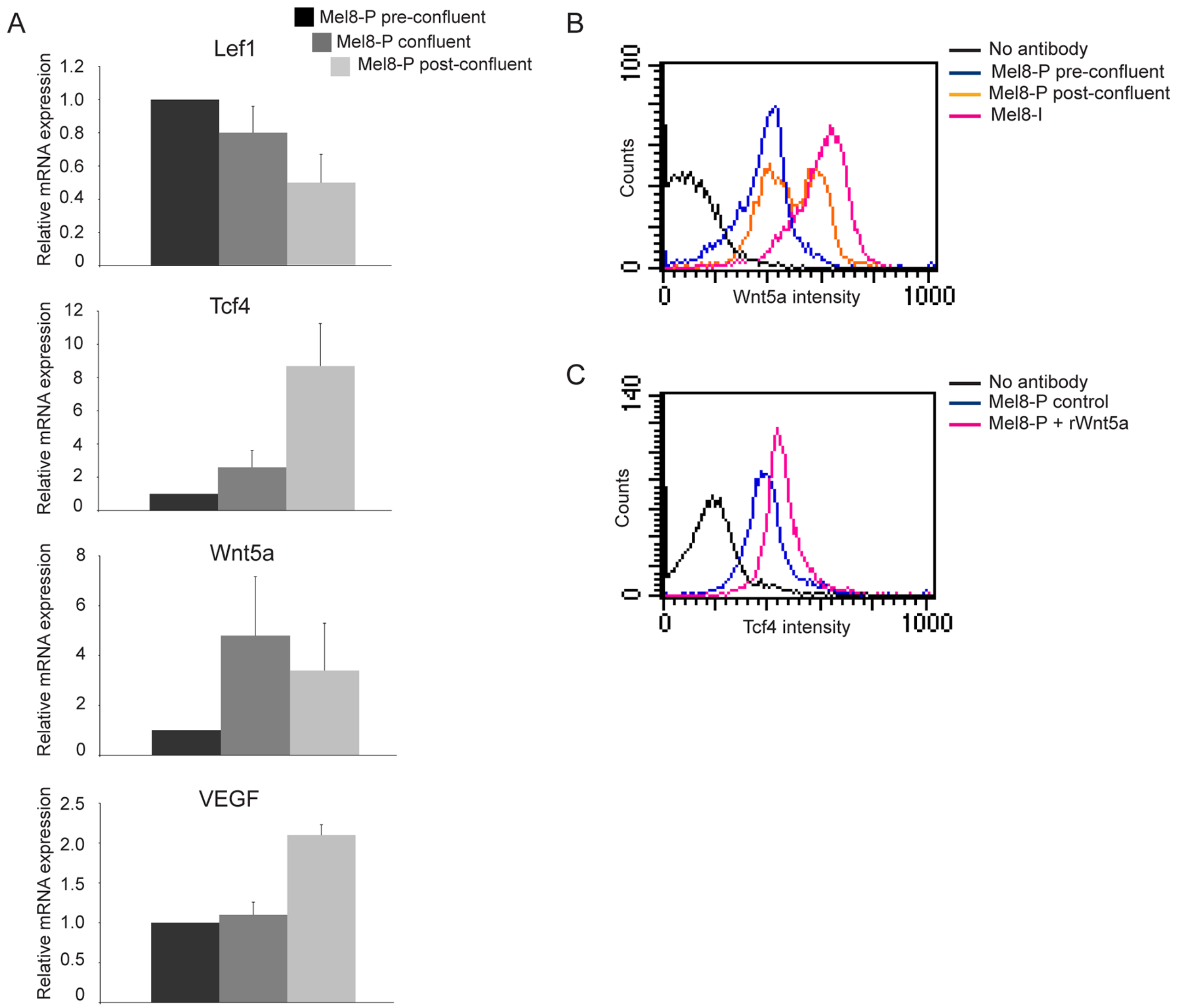

Supplementary Figure S4: Modification in the phenotypic behaviour of Mel8 in relation to cell confluency. Analysis of LEF1, TCF4, WNT5a and VEGF mRNA in Mel8 at different state of confluence **A.** Values represent the means ± SD of fold-increase considering arbitrarily the pre-confluent subpopulation as control (value=1). Flow cytofluorimetric analysis **B.** of Wnt5a level of expression in Mel8-P at pre and post confluence and in floating Mel8-I. Flow cytofluorimetric analysis **C.** of Tcf4 level of expression in adherent Mel8-P treated for 24 hours with the recombinant full lenght WNT5a (rWNT5a, 0.1 µg/ml).

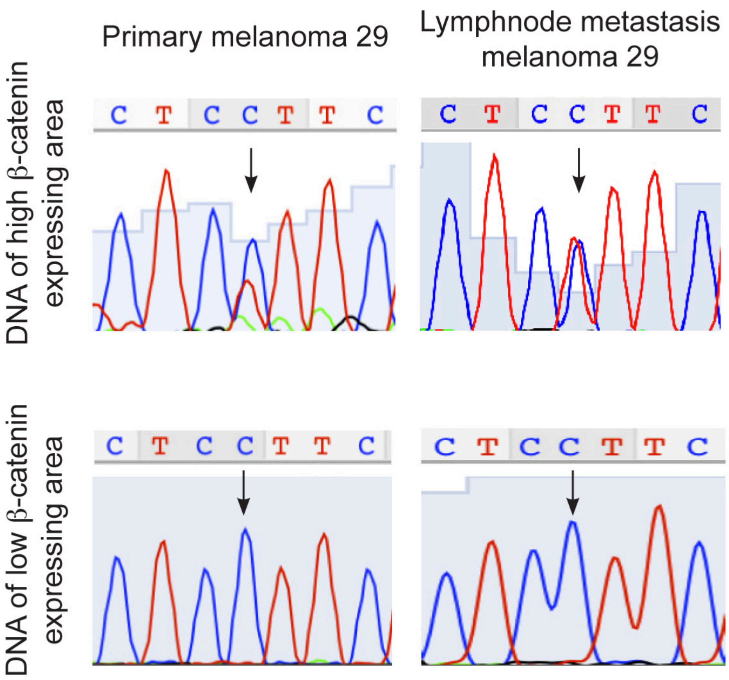

Supplementary Figure S5: Sequence analysis of β-catenin gene in primary lesion and lymphnode metastasis of melanoma 29. DNA sequence of exon 3 of CTNNB1 gene from primary and lymphnode metastasis of melanoma 29. Laser capture microdissection was used to select areas expressing high and low β-catenin from the histological sections.

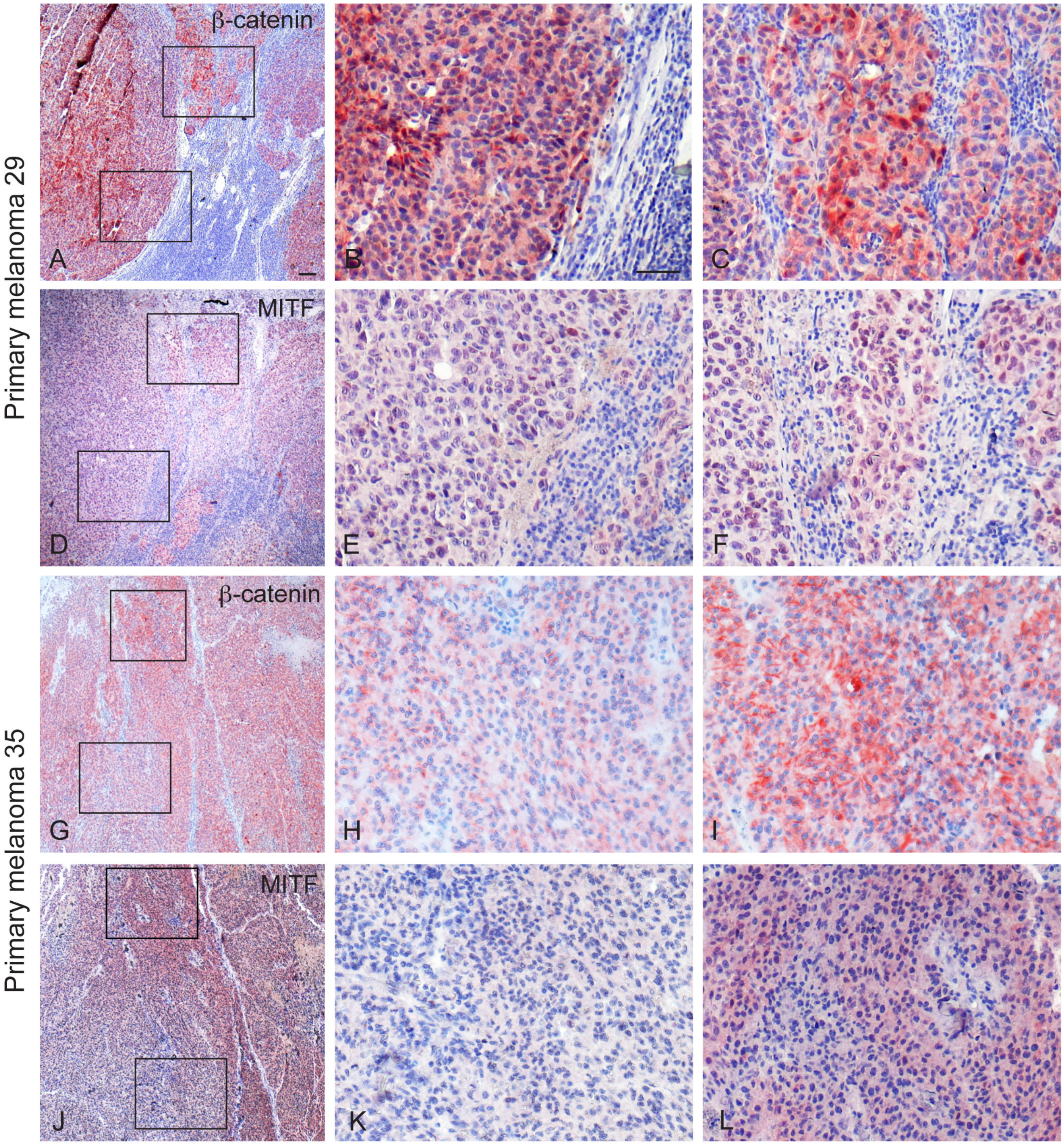

Supplementary Figure S6: Immunohistochemical analysis of β-catenin and MITF expression. Serial sections of paraffin-embedded primary melanomas of patients 29 and 35 were analyzed for β-catenin **A-C G-I.** and MITF **D-F J-L.** expression. Positive MITF immunolabelling was mainly localized in tumour areas presenting high β-catenin reactivity. Scale bar: A,D,G,J: 100 μm; B,C,E,F,K,L: 50 μm

Supplementary Table S1: List of primers used for real-time PCR experiments

Target Gene	Forward primer	Reverse primer	RefSeq
AXIN2	5′-GGAGCGGTGTTTGAGGAG-3′	5′-GCCAAGACAGTTCACAAGAG-3′	NM_004655
BCL2	5′-GACAGAGGATCATGCTGTACTT-3′	5′-CCTTGGCATGAGATGCAGGA-3′	NM_000633
CCND1	5′-AATGACCCCGCACGATT-3′	5′-GCACAGAGGGCAACGAAGG-3′	NM_053056
c-MYC	5′-TCAAGAGGCGAACACACAAC-3′	5′-GGCCTTTTCATTGTTTTCCA-3′	NM_002467
CD44	5′-GGACAAGTTTTGGTGGCACG-3′	5′-TCCGTCCGAGAGATGCTGTA-3′	NM_000610
DKK1	5′-GCCTCAGGATTGTGTTGTG-3′	5′-TGTGAAGCCTAGAAGAATTACTG-3′	NM_012242
CDH1	5′-GAACGCATTGCCACATACAC-3′	5′-ATTCGGGCTTGTTGTCATTC-3′	NM_004360
FN1	5′-CCTCGAAGAGCAAGAGGCAG-3′	5′-GCTTCAGGTTTACTCTCGCA-3′	NM_212482
GAPDH	5′-TGCACCACCAACTGCTTAGC-3′	5′-GGCATGGACTGTGGTCATGAG-3′	NM_002046
IL-1α	5′-CGCCAATGACTCAGAGGAAGA-3′	5′-AGGGCGTCATTCAGGATGAA-3′	NM_000575
IL-1β	5′-CTGAGCTCGCCAGTGAAATG-3′	5′-TTTAGGGCCATCAGCTTCAAA-3′	NM_000576
IL-6	5′-AGCCACTCACCTCTTCAGAACG-3′	5′-GGTTCAGGTTGTTTTCTGCCAG-3′	NM_000600
CXCL8	5′-CTTGGCAGCCTTCCTGATTTC-3′	5′-TTCTGTGTTGGCGCAGTGTG-3′	NM_000584
LEF1	5′-TTCCTTGGTGAACGAGTC-3′	5′-GGGTCCCTTGTTGTAGAG-3′	NM_001130714
MITF	5′-ATGGACGACACCCTTTCTC-3′	5′-GGAGGATTCGCTAACAAGTG-3′	NM_006722
MMP1	5′-CTGGCCACAACTGCCAAATG-3′	5′-CTGTCCCTGAACAGCCCAGTACTTA-3′	NM_002421
MMP2	5′-TCTCCTGACATTGACCTTGGC-3′	5′-CAAGGTGCTGGCTGAGTAGATC-3′	NM_004530
MMP3	5′-ATTCCATGGAGCCAGGCTTTC-3′	5′-CATTTGGGTCAAACTCCAACTGTG-3′	NM_002422
MMP7	5′-TGAGCTACAGTGGGAACAGG-3′	3′-TCATCGAAGTGAGCATCTCC	NM_002423
MMP9	5′-GACGATGACGAGTTGTGGTCC-3′	5′-GGCCCTCGAAGATGAAGGGG-3′	NM_004994
PAX3	5′-CGTGCCGTCAGTGAGTTC-3′	5′-CCTCTGCCTCCTTCCTCTC-3′	NM_181461
CDKN2A	5′- GAGCAGCATGGAGCCTTC-3′	5′-CATCATCATGACCTGGATCG-3′	NM_000077
SOX9	5′-CACGCTGACCACGCTGAG-3′	5′-TGCTGCTGCTCGCTGTAG-3′	NM_000346
BIRC5	5′-GCGTAAGATGATGGATTTGA-3′	5′-GGAACAGCCGAGATGACC-3′	NM_001168
WIF	5′-TACGAAGCCAGCCTCATAC-3′	5′-TGTCGGAGTTCACCAGATG-3′	NM_007191
WISP1	5′-GGCTCTTCCTTGAATCTTCTCC-3′	5′-CCAGGGCACCACTCTCAG-3′	NM_003882
WNT5A	5′-AGCACGACGAAGCAACCTTG-3′	5′-GCCCTCTCCACAAAGTGAACAG-3′	NM_003392
VEGF	5′-GTTGACCTTCCTCCATCC-3′	5′-TTCTCTGCCTCCACAATG-3′	NM_001171630
TCF4	5′-AAGACTGGATGATGCTATTC-3′	5′-CTGATACTCTGGACACTGAAG-3′	NM_003199
SOX2	5′-CTGCAGCTGAAATTTAGGACAGT-3′	5′-AGACCACAGAGATGGTTCGC-3	NM_003106
